# 

**Published:** 1858-07

**Authors:** 


### LIST OF COLLABORATORS.

S. G. Armor, M.D., Professor of Pathology and Clinical Medicine, Missouri Med. College.

John L. Atlee, M.D., President Pennsylvania State Medical Society.

Walter F. Atlee, M.D., Philadelphia.

Robert G. Barclay, M.D., Jerusalem, Syria.

John Bell, M.D., Philadelphia, formerly Professor of Medicine, Medical College of Ohio.

T. S. Bell, M.D., Professor of Medicine, University of Louisville.

S. M. Bemiss, M.D., Louisville, Ky.

L. P. Blackburn, M.D., Natchez, Miss.

George C. Blackie, M.D., Professor of Botany, University of Nashville.

G. C. Blackman, M.D., Professor of Surgery, Medical College of Ohio.

W. S. Bowen, M.D., New York.

J. L. Bobbs, M.D., formerly Professor of Anatomy, Indianapolis.

W. M. Boling, M.D., Montgomery, Ala., formerly Professor of Midwifery, Transylvania University, Lexington, Ky.

N. Bozeman, M.D., Montgomery, Ala.

J. T. Bradford, M.D., Augusta, Ky.

John H. Brinton, M.D., Lecturer on Operative Surgery, Philadelphia.

W. S. Chipley, M.D., Professor of Medicine, Transylvania University.

A. Clapp, M.D., New Albany, Ia.

D. B. Cliffe, M.D., Franklin, Tenn.

J. Da Costa, M.D., Lecturer on the Practice of Medicine at the Philad. Med. Association.

M. Dupierris, M.D., Havana, Cuba.

W. F. Edgar, M.D., United States Army.

S. C. Farrar, M.D., Jackson, Miss.

C. S. Fenner, M.D., Memphis, Tenn.

Austin Flint, M.D., Professor of Clinical Medicine and Pathology, University of Buffalo.

Charles Frick, M.D., Baltimore.

W. Y. Gadbury, M.D., Benton, Miss.

O. C. Gibbs, M.D., Chautauque County, New York.

Dr. J. P. Hall, Glasgow, Ky.

John Hardin, M.D., Professor of Obstetrics, Kentucky School of Medicine, Louisville.

EDWARD HARTSHORNE, M.D., One of the Surgeons to Wills Hospital for Diseases of the Eye, Philadelphia.

E. B. HASKINS, M.D., Clarksville, Tenn., late President of the Tennessee State Medical Society.

C. A. HENTZ, M.D., Marianna, Florida.

ADDINELL HEWSON, M.D., One of the Surgeons to Wills Hospital for Diseases of the Eye, Philadelphia.

SAMUEL L. HOLLINGSWORTH, M.D., late Editor of the Medical Examiner, Philadelphia.

WILLIAM A. HAMMOND, M.D., United States Army.

WILSON JEWELL, M.D., Philadelphia, President of the Quarantine and Sanitary Convention.

WM. V. KEATING, M.D., Lecturer on Obstetrics and the Diseases of Women, in the Philadelphia Medical Association.

JOHN G. KERR, M.D., Canton, China.

G. A. KUNKLER, M.D., Madison, Ia.

BEN. LOGAN, M.D., Shreveport, La.

A. LOPEZ, M.D., Mobile, Ala., formerly President of the Alabama State Medical Society.

GEORGE R. MOREHOUSE, M.D., Philadelphia.

S. WEIR MITCHELL, M.D., Lecturer on Physiology at the Philad. Med. Association.

B. I. RAPHAEL, M.D., New York.

J. C. REEVE, M.D., Dayton, Ohio.

L. C. RIVES, M.D., formerly Professor of Midwifery, Medical College of Ohio.

J. LAWRENCE SMITH, M.D., Professor of Chemistry, University of Louisville.

W. C. SNEED, M.D., Frankfort, Ky., President Kentucky State Medical Society.

C. H. SPILMAN, M.D., Harrodsburg, Ky., late President of Kentucky State Medical Society.

GEO. SUTTON, M.D., Aurora, Ia.

W. L. SUTTON, M.D., Georgetown, Ky., formerly President Kentucky State Medical Society.

HORATIO R. STORER, M.D., formerly one of the Physicians to the Boston Lying-in Hospital, Boston, Mass.

S. THOMPSON, M.D., Albion, Ill.

E. WILLIAMS, M.D., Cincinnati, Ohio.

### Bi-Monthly Bibliographical Record.

AMERICAN.

Contributions to Operative Surgery and Surgical Pathology. By J. M. Carnochan, M.D., Professor of Surgery in the New York Medical College. No. 1. 4to., pp. 32. Philadelphia, 1858: Lindsay & Blakiston. (From the publishers.)

Silver Sutures in Surgery: the Anniversary Discourse before the New York Academy of Medicine. By J. Marion Sims, M.D. Published by the Academy. pp. 79. New York, 1858. (From the author.)

Transactions of the Medical Society of the State of New York. 8vo., pp. 665. 1858.

Fifteenth Annual Report of the Managers of the New York State Lunatic Asylum. 1858.

Case of Diabetes Mellitus. By Joseph Jones, M.D., etc. 8vo., pp. 25. Reprint. (From the author.)

Quarterly Summary of the Transactions of the College of Physicians of Philadelphia. pp. 49., 8vo. 1858.

Transactions of the Eighth Annual Meeting of the Medical Society of the State of North Carolina. 8vo., pp. 90. 1857.

Proceedings of the American Pharmaceutical Association, at the Sixth Annual Meeting held in Philadelphia, September, 1857. 8vo., pp. 178.

FOREIGN.

Ergebnisse und Studien aus der Medicinischen Klinik in Bonn. Von Dr. M. E. A. Nauman, Prof. Leipzig, 1858. pp. 408.

The Ganglionic Nervous System; its Structure, Functions, and Diseases. By G. Davey, M.D. London, 1858. pp. 309.

The Cause of the Coagulation of the Blood. By B. W. Richardson, M.D. London, 1858. pp. 466.

A Catechism on the Physiology and Philosophy of Body, Sense, and Mind. By J. W. Jones, F.R.S. London, 1858. pp. 124.

Gleet; its Pathology and Treatment. By Henry Dick, M.D. London, 1858. pp. 88.

Elements of Practical Midwifery. By C. Waller, M.D. Fourth Edition. London, 1858. pp. 153.

On the Malformations of the Human Heart. By Thomas B. Peacock, M.D. London, 1858. pp. 142.

Dislocations and Fractures. By Joseph Maclise, F.R.C.S. Fasciculus II. London, 1858.

The Physiological Anatomy and Physiology of Man. By Robert Bentley Todd, M.D., F.R.S., and William Bowman, F.R.S. Part IV.; Section Second. 8vo., pp. 241. J. W. Parker & Son: London, 1857.

A System of Practical Surgery. By William Ferguson, F.R.S. Fourth Edition. 12mo., pp. 833. Churchill: London, 1857.

A Practical Treatise on the Diseases of Infancy and Childhood. By T. H. Tanner, M.D., F.R.S., etc. London: Renshaw, 1858.

Leçons sur le Chancre, professées par le Docteur Ricord; redigées et publiées par Alfred Fournier, Interne de l'Hôpital du Midi. 8vo., pp. 351. Adrien Delahaye: Paris, 1858.

The Diseases, Injuries, and Malformations of the Rectum. By T. J. Ashton, Surgeon to the Blenheim Street Dispensary. 8vo. London: Churchill, 1858.

On Some of the More Obscure Forms of Nervous Affections; their Pathology and Treatment. By Harry W. Lobb, L.S.A. 8vo. London: Churchill, 1858.

A Manual of Psycological Medicine. By John E. Bucknill, M.D., and Daniel H. Tuke, M.D. 8vo. Churchill: London, 1858.

A Manual of Medical Jurisprudence. By Alfred S. Taylor, M.D., etc. Sixth edition. 8vo. Churchill: London, 1858.

A Sketch of the Principles and Practice of Subcutaneous Surgery. By William Adams, F.R.C.S. London: Churchill, 1858.

Of Nature and Art in the Cure of Disease. By Sir John Forbes, M.D., etc. 12mo., pp. 261. Republished from the Second London Edition by S. S. & W. Wood: New York, 1858. (From the publishers.)

Mind and Matter; or Physiological Inquiries. By Sir Benjamin Brodie, Bart., etc. 12mo., pp. 279. Republished, with additional Notes by an American Editor, by S. S. & W. Wood: New York, 1858. (From the publishers.)

Clinical Lectures on the Principles and Practice of Medicine. By John Hughes Bennett, M.D., F.R.C.S., Professor of Medicine in the University of Edinburgh. Second Edition. 8vo., pp. 951. Samuel S. & Wm. Wood: New York, 1858. (From the publishers.)

An Essay on Wasting Palsy. By William Roberts, M.D., Physician to the Manchester Royal Infirmary. 8vo. Churchill: London, 1858.

Congrès d'Ophthalmologie de Bruxelles. Compte-Rendu, publié au nom de Bureau. Par le Docteur Warlomont. Session de 1857. 8vo., pp. 492. Victor Masson: Paris, 1858.

Evil Results of Over-Feeding Cattle. By Frederick James Gant, M.R.C.S., etc. pp. 39. Churchill: London, 1858. (From the author.)

